# Bentall procedure: quarter century of clinical experiences of a single surgeon

**DOI:** 10.1186/s13019-016-0418-y

**Published:** 2016-01-22

**Authors:** Kálmán Benke, Bence Ágg, Lilla Szabó, Bálint Szilveszter, Balázs Odler, Miklós Pólos, Chun Cao, Pál Maurovich-Horvat, Tamás Radovits, Béla Merkely, Zoltán Szabolcs

**Affiliations:** Heart and Vascular Center, Semmelweis University, H-1122 Városmajor str. 68, Budapest, Hungary; Department of Pulmonology, Semmelweis University, Budapest, Hungary; Hungarian Marfan Foundation, Budapest, Hungary; MTA-SE Lendület Cardiovascular Imaging Research Group, Heart and Vascular Center, Semmelweis University, Budapest, Hungary

**Keywords:** Bentall procedure, Aortic root reconstruction, Cardiac surgery, Single surgeon experience

## Abstract

**Background:**

We retrospectively analyzed 25 years of experiences with the button Bentall procedure in patients with aortic root pathologies. Even though this procedure has become widespread, there are only a few very long term follow-ups available in the clinical literature, especially regarding single surgeon results.

**Methods:**

Between 1988 and 2013, a total of 147 patients underwent the Bentall procedure by the same surgeon. Among them there were 62 patients with Marfan syndrome. At the time of the surgery the mean age was 46.5 ± 17.6 years. The impact of surgical experience on long-term survival was evaluated using a cumulative sum analysis chart.

**Results:**

The Kaplan-Meier estimated overall survival rates for the 147 patients were 91.8 ± 2.3 %, 84.3 ± 3.1 %, 76.3 ± 4.9 % and 59.5 ± 10.7 % at 1,5,10 and 20 years, respectively. Multivariate Cox regression analysis identified EuroSCORE II over 3 % (OR 4.245, 95 % CI, 1.739–10.364, *p* = 0.002), acute indication (OR 2.942, 95 % CI, 1.158–7.480, *p* = 0.023), use of deep hypothermic circulatory arrest (OR 3.267, 95 % CI, 1.283–8.323, *p* = 0.013), chronic kidney disease (OR 6.865, 95 % CI, 1.339–35.189, *p* = 0.021) and early complication (OR 3.134, 95 % CI, 1.246–7.883, *p* = 0.015) as significant risk factors for the late overall death. The survival rate for freedom from early complication was 94.3 ± 2.2 %, 88.0 ± 3.3 %, 82.9 ± 4.7 % and 69.2 ± 8.4 % at 1,5,10 and 20 years. The main pathological findings of the aortic wall were cystic medial degeneration in 75 %, fibrosis in 6 %, atherosclerosis in 13 % and no pathological alteration in 6 % of the samples. The overall survival rate was significantly lower in patients operated in first 15 years compared to patients operated in the last decade (log-rank *p* = 0.011).

**Conclusion:**

According to our long-term follow-up the Bentall operation provides an appropriate functional result by resolving the lesions of the ascending aorta. Based on our results, 25–30 operations done is necessary to gain such a level of confidence and experince to aquire better results on long-term survival. In addition, we discussed that there were no co-morbidities affecting on the survival of Marfan patients and prophylactic aortic root replacement ensures a longer survival among patients with Marfan syndrome.

## Background

Aortic root replacement procedures, which include the modified version of the original Bentall [1] operation, the “button Bentall procedure”, which is an open technique for reattaching the coronary ostia. Although this procedure has become widespread, there are only a few very long term follow-ups available in the clinical literature. We evaluated the long-term clinical outcomes and sought to determine the independent predictors of long-term mortality for the button Bentall procedure in 147 patients [2]. We analyzed the independent risk factors of long-term mortality in a subdivided Marfan population too. Furthermore, there has been no study published yet about experience and results of a single surgeon in Bentall procedure.

## Methods

### Patients’ characteristics

We retrospectively analyzed 25 years of experience with Bentall procedure. Between 1988 and 2013, a total of 147 patients who underwent aortic root reconstruction at the Heart and Vascular Center, Semmelweis University. Of these patients, 111 (75 %) were male and 36 were female (25 %). 62 patients were with Marfan syndrome (42 %) among them and the diagnosis of the syndrome was verified in every case with the use of the original and later the revised Ghent criteria [3]. Subanalysis of the Marfan syndrome group was performed.

An electronic Aortic Root Reconstruction Registry database has been established which includes demographics, types of indications, comorbidities, procedure specifications and follow-up informations. Medical records and patient’s history were used to identify comorbidities. Patients’ follow-ups included clinical examination, computed tomography scans and transthoracic echocardiography, and they were treated by the same surgeon. During the studied period, Tirone David valve-sparing procedures were performed in 27 patients. These patients were excluded from the study.

The mean patient age at the time of the operation was 46.3 ± 17.5 years (range, 8–78 years), 8 (5 %) patients were older than 70 years. We measured body parameters of patients (height and weight), and calculated the Body Mass Index. The mean BMI was 25.6 ± 5.6 kg/m^2^. We found that 80 patients had hypertension (54 %), 11 patients had diabetes mellitus (7 %) and 6 had hyperlipidaemia (4 %). 21 patients were suffering from coronary artery disease (14 %) at the time of the operation, 4 from chronic kidney disease (3 %) and 7 patients had cerebrovascular accident (5 %). The cardiovascular functional status was determined according to the New York Heart Association (NYHA). EuroSCORE II was calculated in all patients according to the EuroSCORE II protocol [4]. The histology of the aortic wall and valve were available in 114 patients (78 %). The main indications of the operation were annuloaortic ectasia (61 %), acute aortic dissection (16 %) and chronic aortic dissection (23 %). Preoperative variables are depicted in Table 1.

**Table 1:** Preoperative characteristics

Preoperative variables	n (%)
Patients	147
Age (yrs) ± SD	46.3 ± 17.5
Male/Female	111/36 (75/25)
Hypertension	80 (54)
Diabetes mellitus	11 (7)
Coronary artery disease	21 (14)
HLP	6 (4)
BMI (kg/m^2^)	25.6 ± 5.6
Cerebrovascular accident	7 (5)
Chronic kidney disease	4 (3)
NYHA class	1.3 ± 0.6
EuroSCORE II (%)	4 [2–6]
Aorta ascendens diameter (mm)	61.7 ± 16.3
Marfan syndrome	62 (42)
Previous Cardiac Operation	6 (4)
Grade of aortic regurgitation	2.8 ± 1.5
Ejection fraction (%) [before OP / after OP]	49.6 ± 8.3 / 54.33 ± 11.17
All-cause death	29 (20)

### Surgical technique

The operation was performed via median sternotomy, and cardiopulmonary bypass was applied by cannulating the ascending aorta, aortic arch, femoral artery, or axillar artery, and the right atrium. Myocardial protection was provided by anterograde, retrograde, or simultaneously anterograde and retrograde intermittent cold hyperkalemic blood cardioplegia. Deep hypothermic circulatory arrest (DHCA) was used in 28 patients (19 %). The coronary buttons were excised with the aortic wall patch and mobilized to facilitate reimplantation. The proximal anastomosis was implemented with pledgeted interrupted sutures. The distal, grafto-aortic anastomosis was accomplished with continuous sutures and the coronary button anastomoses were also performed with continuous sutures.

St. Jude composite graft (St. Jude Medical, Inc., St.Paul, MN, USA) was used in 101 patients; Carbomedics composite graft (Carbomedics, Austin, TX, USA) in 16 patients; Carbomedics Carbo-Seal conduit (Sorin, Milano, Italy) in 18 patients; Vascutek Gelweave composite graft (Vascutek, Scotland, UK) in 5 patients; Hancock bioprosthesis (Medtronics, Minneapolis, Minnesota, USA) with Vaskutek straight graft in 5 patients; and Shelhigh bioconduit (Shelhigh, Union, NJ, USA) in 2 patients. The 7 bioprosthetic valve and graft were compiled together by the surgeon during operation.

The concomitant procedures were performed in 39 patients (27 %); included mitral valve surgery in 11, coronary artery bypass grafting (CABG) in 12, hemi- and total arch replacement in 10, pacemaker implantation in 2, and others in 4 patients. Operative data are described in Table 2.

**Table 2:** Operative data

Operative data	n (%)
Aortic pathology	
Annuloaortic ectasia	89 (61)
Dissection (acute/chronic)	24/34 (16/23)
Bicuspid aortic valve	11 (7)
Implanted valve type	
Mechanical valve	140 (95)
Bioprosthetic valve	7 (5)
Composite valve size (mm)	25.9 ± 1.7
Comcomitant cardiac procedures	
Mitral valve surgery	11 (7.5)
Coronary artery bypass	12 (8)
Total arch replacement	10 (7)
Pacemaker implantation	2 (1)
Others	4 (2)
Cardiopulmonary bypass	
Operation time (min)	245 [210–305]
Cardiopulmonary bypass time (min)	155 [130–185]
Aortic cross-clamp time (min)	113 [100–137]
DHCA use	28 (19)
Pharyngeal temperature (˚C)	25.6 ± 6.1
Timing of operation	
Emergency	24 (16)
Urgency	34 (23)

### Follow-up and statistical analysis

We selected all-cause mortality as the endpoint. No patient was lost during the follow-up period. Death was detected from death records of the Hungarian National Health Insurance Fund, which provided accurate mortality data for every patient. Follow-up period for the overall survival was measured from the date of the operation to the date of death, or of last contact alive. Follow-up ended on October 2013. 118 patients (80 %) of survivors had completed follow-up. The mean length of the follow-up periods was 84 ± 56 months.

All continuous variables were expressed as mean ± SD or median with interquartile ranges, whereas categorical variables were expressed as percentage. The Shapiro-Wilk test was used to check the normality of the data before further analysis. For the analysis of the data we used Student’s *t*-test, Mann–Whitney *U*-test and *χ*2 test. Univariate and multivariate analysis of predictors for mortality were performed using a Cox regression model evaluate the association between independent risk factors and mortality. Survival curves were created using the Kaplan-Meier method and compared with the log-rank test. Multivariate logistic regression analysis was used to identify the risk factors of early complication.

We used univariate and multivariate Cox regression analysis to evaluate the effect of comorbidities on mortality. Multivariate Cox regression identified the independent risk factors of long-term mortality after Bentall procedure. The determination of risk factors was carried out by selecting variables with *p* < 0.10 from univariate Cox regression, and further examined our data with multivariate Cox regression analysis. A *p*-value of <0.05 was considered statistically significant (Table. 4).

The impact of surgical experience on survival was evaluated using a time-adjusted cumulative sum complication chart. The statistical principles were adapted from the comprehensive tutorial by Rogers et al. [5]. Cumulative sum (CUSUM) analysis is defined as Sn = (X_i_ – p0_i_), where X_i_ = 0 for operation without complication and 1 for the presence of early complication, and p0_i_ denotes the predicted probability of the development of early complication within 30 days after surgery. The graph starts at zero, but is incremented by 1- p0_i_ for formation of early complication and decremented by p0_i_ for uncomplicated operation [6]. This graph is very descriptive because it moves upwards if the complication rate increases above the risk model predicted results, moves downwards if the rate decreases and oscillates around zero performance which is consistent with predicted risks thus is considered as acceptable [6]. Data were stored in Aortic Root Reconstruction Register and analysed with the SPSS statistical program (version 20.0,Chicago, IL, USA).

## Results

### Early outcomes

The operative mortality was 2 %. The overall early mortality rate, defined as death within 30 days of initial hospitalization, was 3.4 % (5/147). Causes of early death were low cardiac output syndrome (*n* = 1), diffuse hypoxic brain damage (*n* = 2), ventricular arrhytmia (*n* = 1) and excess bleeding in 1 patient. Early complications included postoperative resternotomy for bleeding (*n* = 9), atrial and ventricular arrhytmias (*n* = 13), renal failure needing hemodialysis (*n* = 1), cerebral infarction (*n* = 2) and pericardial tamponade (*n* = 1) (Table 3).

**Table 3:** Causes of complications

Complications	n (%)
Intraoperative complications	
Bleeding	7 (5)
Arrythmia	3 (2)
Early postoperative complications	
Bleeding	9 (7)
Diffuse cerebral hypoxia	2 (1)
Pericardial tamponade	1 (0.7)
Renal failure needed hemodialysis	1 (0.7)
Arrythmia	13 (9)
Late postoperative complications	
Bleeding	1 (0.7)
Arrythmia	2 (1)

Multivariate logistic regression analysis revealed, that NYHA class of III and IV (OR 9.2, 95 % CI, 0.972–87.240, *p* = 0.050), dissection (OR 6.817, 95 % CI, 1.392–33.393, *p* = 0.018), concomitant CABG surgery (OR 15.722, 95 % CI, 3.087–80.064, *p* = 0.001) and concomitant mitral valve surgery (OR 5.207, 95 % CI, 0.987–27.480, *p* = 0.049) were the independent risk factors for early complication, which defined as possible life-threathening complication within 30 days of initial hospitalization (Table 5).

### Long-term results

The mean survival was 190 ± 10.3 months (IQ range:170–210 months). There have been 23 late deaths (death after one year), whereof 18 cardiac-related (78 %) and 5 non-cardiac-related (22 %) deaths occured. The most common causes of cardiac-related death were aneurysm rupture of descending aorta, sudden cardiac death, congestive heart failure, CVA and low cardiac output syndrome.

**Fig. 1:**
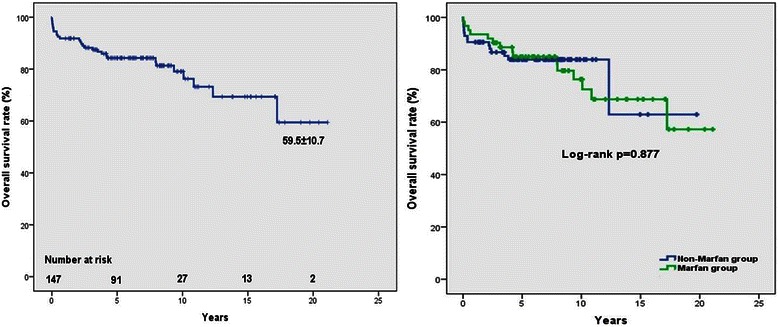
Kaplan-Meier estimated survival curves. Actual survival of all patients, at the mean follow-up time of 190 ± 10.3 months, following the Bentall procedure (**a**). Actual survival of Marfan and non-Marfan syndrome patients (**b**)

Kaplan-Meier estimated overall survival rates for the 147 patients (including deaths occured at the initial hospitalization) were 91.8 ± 2.3 %, 84.3 ± 3.1 %, 76.3 ± 4.9 % and 59.5 ± 10.7 % at 1,5,10 and 20 years, respectively (Fig. 1a). Multivariate Cox regression analysis identified EuroSCORE II over 3 % (OR 4.245, 95 % CI, 1.739–10.364, *p* = 0.002), acute indication (OR 2.942, 95 % CI, 1.158–7.480, *p* = 0.023), DHCA use (OR 3.267, 95 % CI, 1.283–8.323, *p* = 0.013), chronic kidney disease (OR 6.865, 95 % CI, 1.339–35.189, *p* = 0.021), and early complication (OR 3.134, 95 % CI, 1.246–7.883, *p* = 0.015) as significant risk factors for the late overall death (Table 6).

### Influence of Marfan syndrome on survival

We have separated the patients into two groups: patients with Marfan syndrome (Marfan group), and patients without Marfan syndrome (non-Marfan group) (Table 4). Multivariate regression analysis of all patients’ data revealed, that EuroSCORE II over 3 % (OR 5.612, 95 % CI, 1.761–17.884, *p* = 0.004) and acute indication (OR 6.391, 95 % CI, 1.373–29.750, *p* = 0.018) were the independent risk factors for mortality (Table 5). Among the patients with Marfan syndrome, the overall survival rate did not reach statistical significance (log rank *p* = 0.877) compared to the non-Marfan group (Fig. 1b).

**Table 4:** Risk factor analysis for late overall death

Variables	Patients (*n* = 147)		Patients with Marfan syndrome (*n* = 62)	
	Univariate	Multivariate	Univariate	Multivariate
Age	0.821		0.584	
Sex (male)	0.097	0.089	0.226	
Hypertension	0.141		0.156	
Diabetes mellitus	0.348		-	
Hyperlipidaemia	0.577		0.787	
BMI > 30	0.448		0.335	
Coronary artery disease	0.167		0.390	
Cerebrovascular accindent	0.505		0.682	
Chronic kidney disease	0.011^a^	0.036^a^	0.683	
EuroSCORE II > 3 (%)	<0.0001^a^	0.002^a^	<0.0001^a^	0.004^a^
Aortic diameter > 60 mm	0.211		0.369	
Bicuspid aortic valve	0.538		-	
Emergency	0.006^a^	0.023^a^	0.006^a^	0.018^a^
Combined mitral valve surgery	0.856		0.547	
Combined CABG surgery	0.062		0.701	
Combined with arch replacement	0.855		0.995	
Ejection fraction < 50 %	0.681		0.252	
LVESD	0.805		0.370	
LVEDD	0.534		0.206	
Dissection	0.035^a^	0.136	0.094	0.476
CPB time	0.002^a^	0.710	0.982	
Operation time > 5 h	0.002^a^	0.224	0.102	
DHCA use	0.004^a^	0.013^a^	0.597	
Aortic cross-clamp time	0.753		0.800	
Early complication	0.035^a^	0.015^a^	0.190	

^a^statistically significant

**Table 5:** Independent predictors of late overall mortality in multivariate cox regression analysis

Variables	Patients (*n* = 147)		
	OR	95 % CI	*P*-value
EuroSCORE II > 3 %	4.245	1.739–10.364	0.002
Emergency	2.942	1.158–7.480	0.023
DHCA use	3.267	1.283–8.323	0.013
Chronic kidney disease	6.865	1.339–35.189	0.021
Early complication	3.134	1.246–7.883	0.015
	Patients with Marfan syndrome (*n* = 62)		
	OR	95 % CI	*P*-value
EuroSCORE II > 3	5.612	1.761–17.884	0.004
Emergency	6.391	1.373–29.750	0.018

### Outcome of dissections

**Fig. 2:**
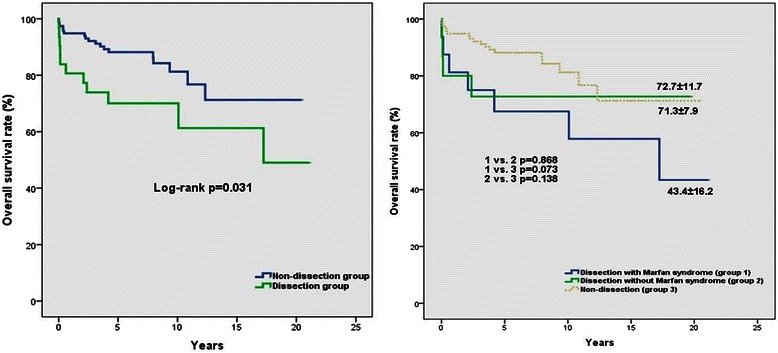
Kaplan-Meier estimated survival curves. Actual survival rate for non-dissection and dissection patients (**a**). Actual survival of dissection with Marfan syndrome (group 1), dissection without Marfan syndrome (group 2) and non-dissection (group 3) patients (**b**)

Overall survival rate differed between the non-dissection group and the dissection group. In the latter group the results were significantly lower according to the log-rank test (*p* = 0.031) (Fig. 2a). However in Marfan syndrome patients with dissection, the overall survival rate tended to decline compared to the dissection group without Marfan syndrome and to the non-dissection group (Fig. 2b).

### Histopathology of the aortic wall and valve

During the operation aortic wall and valve samples were collected and sent for pathological examination. The main pathological findings unearthed were cystic medial degeneration (CMD) in 85 samples (75 %), fibrosis in 7 samples (6 %), atherosclerosis in 15 samples (13 %) and no pathological alteration in 7 samples (6 %). Overall survival rates between the patients with CMD, patients with fibrosis and patients with atherosclerosis were similar (CMD vs. fibrosis log-rank *p* = 0.197; CMD vs. atherosclerosis log-rank = 0.400; fibrosis vs. atherosclerosis log-rank *p* = 0.876) Fig. 2.

### Impact of surgical experience on survival

**Fig. 3:**
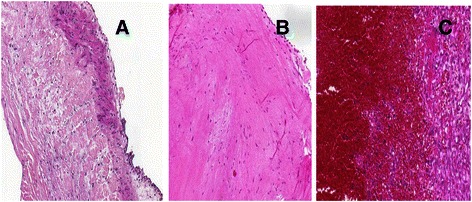
Histological findings of the aortic wall. Cystic medial degeneration (**a**) in 75 %, fibrosis (**b**) in 6 %, atherosclerosis (**c**) in 13 % of the samples

**Fig. 4:**
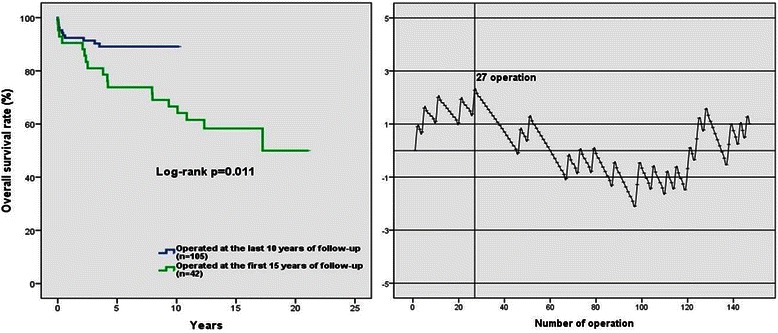
Actual survival of patients operated at the first fifteen years of follow-up and patients operated in the last decade (**a**). The risk-adjusted CUSUM complication curve for the entire follow-up (**b**)

We have compared the first 15 years of results to the last 10 years of results to investigate the influence of surgical experience on survival. From 1998 till the end of 2003 42 patients were operated (29 %). The other 105 patients were operated at the next decade (71 %). The overall survival rate was significantly lower in patients operated in first 15 years compared to patients operated in the last decade (log-rank *p* = 0.011) (Fig. 4a). The time-adjusted CUSUM complication curve for the entire follow-up time is illustrated in (Fig. 3b). The CUSUM curve presents an upward inflection at the first 27 operation, which indicates the major learning curve effect. After that, we observed a downward inflection, indicating better results and the experience of the surgeon with respect to the predicted complication rates. Specially, we observed three complication between operations no. 121 and no. 124. This cluster of complications necessitated a review. We found that these patients were fragile, multimorbid and urgent cases, than the other as evidenced by the higher predicted probability of complication.

## Discussion

The mean survival in our study was 190 ± 10.3 months (IQ range:170–210 months). There are only few papers published about the Bentall procedure which include mean follow-up duration of more than six years (Table 6).

**Table 6:** Previous reports about Bentall operation

Author	Patient group	Follow-up	Early complication rate (%)	Long-term survival at 5/10/20 years (%)
Tae Sik Kim et al. [2]	*n* = 195, 1997–2010, 47 patients with MFS	median, 64 months	48.7	96/90/-
Patel et al. [21]	*n* = 140 Marfan patients, 1997–2006	mean, 114 months	21.5	90/-/-
Gott et al. [20]	*n* = 675 patients with MFS, 1968–1996 10 centers	mean, 80 months	-	84/75/-
Kouchoukos et al. [7]	*n* = 168, 1974–1990, 30 patients with MFS	mean, 81 months	-	61 at 7 years/ 48 at 12 years/-
Joo et al. [11]	*n* = 218, 1982-2010	mean, 108 months	48.1	83/78/-
Benke et al.	*n* = 147, 1988–2013, 62 patients with MFS	mean, 84 months	25.5	84/76/59

In our study, we report that the independent predictors of early complication include NYHA class of III and IV, dissection and concomitant CABG or mitral valve surgery. The poor cardiac condition might often associated with early arrythmias, while aortic dissection is a well-known risk factor of the bleeding events.[7]

Numerous studies have shown different independent risk factors for death after Bentall procedure [8, 9]. Advanced age, dissection, Marfan syndrome, severe ventricular dysfunction, endocarditis, previous cardiac surgery, emergency status, coronary artery disease, poor preoperative New York Heart Association functional class and left ventricle ejection fraction <35 % were known as predictors of early and late death.[8, 9] We observed that late overall death was strongly correlated with EuroSCORE II over 3 %, acute indication, DHCA use, chronic kidney disease and early complication thus all the above mentioned factors seem to be independent risk factors of late mortality. In regard that common causes of late death such as rupture of descendent aorta aneurysm, sudden cardiac death, congestive heart failure, CVA, low cardiac output syndrome are associated with poor NYHA status and complications [8]. We found that in our research operative mortality and long-term survival after Bentall surgery were comperable to that in other studies [2, 8, 10]. With improvements in operative technique and postoperative management years, Marfan syndrome, diabetes, hypertension, bicuspid aortic valve, hyperlipidaemia, coronary artery disease, cerebral vascular accident, concomitant cardiac surgery, longer operation, aortic cross-clamp time and dissection did not prove to be risk factors for mortality in this study.

There are many debates as to whether Marfan syndrome had an influence in long-term survival after the Bentall procedure. Although several papers have been published [2, 8, 11], on the subject regarding the impact of presence of Marfan syndrome on survival rate, but only few of them studied the Marfan group as a separate patient group. Since Marfan patients are usually operated in younger age and with less comorbidities [12], therefore they should be managed as a distinct patient group. We presented the risk factors of mortality of the Marfan patients separately. In our study, EuroSCORE II over 3 % and acute indication were significant predictors of long-term mortality among patients with Marfan syndrome. These results also confirm, that there were no co-morbidities affecting the survival of Marfan patients. However the prevention of the acute, often life-threatening complications – such as aortic dissection – with prophylactic aortic root replacement ensures longer survival among patients with Marfan syndrome [13]. There was a trend for decreased survival of patients with Marfan syndrome in the course of time compared to that of non-Marfan patients (Fig. 1b), but we could not find a significant difference in long-term survival (*p* = 0.877) [14]. In our study acute or chronic dissection was the surgical indication in 58 cases. Overall survival rates among the dissection groups were comparable to other reports [11].

The long-term survival of the cardiac procedures are examined from the perspective of the duration of the operation [8]. In our series, thirty-six (24 %) patients had a longer, than five hours operation time. The risk factors of extended duration operations were NYHA class of III and IV, dissection, concomitant mitral valve surgery and DHCA use. According to these results, poor NYHA status and the extension of the surgical area increases the length of the surgery, thus patients with worse cardiac status (EF:30-40 %) should be treated for preconditioning with inta-aortic balloon pump (IABP) or with 24 h levosimendan infusion preoperatively.

Although we had incomplete data in 33 cases, histological differences has never been investigated in Bentall studies. In our study, distribution of the histological type were cystic medial degeneration (CMD) in 85 samples (75 %), which is also known as the pathological change of the aortic wall in Marfan patients [15]. Furthermore there were fibrosis in 7 samples (6 %), atherosclerosis in 15 samples (13 %) and no pathological changes in 7 samples (6 %). The survival rates were not different compared to each other among patients with abnormal histological evidence. (CMD vs. fibrosis log-rank *p* = 0.197; CMD vs. atherosclerosis log-rank = 0.400; fibrosis vs. atherosclerosis log-rank *p* = 0.876) (Fig. 2).

During the cardiothoracic training surgeons need to gain experience in Bentall procedure. Hence the surgeon launches on a ‘learning curve’, and unfortunately, his/her patients may possibly be at a higher risk [6]. The traditional way of the surgical audit with retrospective analysis of outcome data and statistical testing is an appropriate method to estimate the learning curve. However, when this is not the case the CUSUM curve is more suitable for this kind of analysis [16]. Hence several publications have used the CUSUM method to asses surgical results in cardiac surgery [16, 17]. However we found no data about the learning curve of the button Bentall procedure. In this study single surgeon results were demonstrated, therefore we could apply the time adjusted cusum complication curve to this case (Fig. 4b). At the beginning the CUSUM curve starts with an upward inflection, which indicates the major learning curve effect. Based on our findings after 27 operations the risk of the early complications started to reduce. This reflects according to our findings, that approximately 25–30 operations are necessary to aquire better results on survival. After that, we observed a downward inflection, indicating better results and an increase in the experience of the surgeon with respect to the predicted complication rates. Subsequently, when the surgeon felt confident with this procedure, he started to consider those patients in worse condition suitable for Bentall surgery and this may have resulted in a small assessment of the curve (Fig. 4b).

Additionally, after comparing the first fifteen years of surgical results to the last decade the overall survival rate we found that the results were significantly lower in first 15 years operated group than the last 10 years operated group (log-rank *p* = 0.011) (Fig. 4a).

Our paper has some limitations that are unaviodable in a retrospective study. Observational data do not provide casual evidence. After fifteen years of observation time only 10 % of of patients still at risk which is a limitation. Due to incomplete data collection, several important variables, such as ejection fraction after surgery and pharyngeal temperature, were omitted in the statistical analysis. The histology of the aortic wall could not be included in logistic regression analysis due to the missing 33 samples. In our study only two pseudoaneurysm formations was observed at the graft anastomosis sites [18, 19], but none of them were Marfan patient. The estimation of odds ratios, with regard to chronic kidney disease (*n* = 4) and NYHA class of III and IV in Marfan patients (*n* = 2), could not be carried out in logistic regression analysis due to the low number of events.

## Conclusion

In summary, as this study was performed from the data of one surgeon, gives us the opportunity to describe the learning curve of the button Bentall procedure and follow the results through his course of carrier. In our series, 25–30 operations gave the surgeon confidence and experince to aquire better results on long-term survival. In addition, we discussed that there were no co-morbidities affecting on the survival of Marfan patients and prophylactic aortic root replacement ensures a longer survival among patients with Marfan syndrome. Finally, upon the statistical results we discussed histological changes of the aortic wall, which has never been described in long-term button Bentall follow-ups.

## Abbreviations

MFS: Marfan syndrome
CUSUM: Cumulative sum
DHCA: Deep hypothermic circulatory arrest
CMD: Cystic medial degeneration
BMI: Body mass index
NYHA: New York Heart Association
CABG: Coronary artery bypass graft
IQ: Interquartile
CVA: Cerebral vascular accident
SD: Standard deviation
LVESD: Left ventricular end systolic diameter
LVEDD: Left ventricular end diastolic diameter
CPB: Cardiopulmonary bypass
IABP: Intra-aortic balloon pump

## References

[1] Bentall H, De Bono A (1968). A technique for complete replacement of the ascending aorta. Thorax.

[2] Kim TS, Na C-Y, Sam Sae O, Kim JH (2013). Long-term mortality and morbidity after Button Bentall operation. J Card Surg.

[3] Loeys BL, Dietz HC, Braverman AC, Callewaert BL, De Backer J, Devereux RB (2010). The revised Ghent nosology for the Marfan syndrome. J Med Genet.

[4] Nashef SA, Roques F, Sharples LD, Nilsson J, Smith C, Goldstone AR (2012). EuroSCORE II. Eur J Cardiothorac Surg.

[5] Rogers CA, Reeves BC, Caputo M, Ganesh JS, Bonser RS, Angelini GD (2004). Control chart methods for monitoring cardiac surgical performance and their interpretation. J Thorac Cardiovasc Surg.

[6] Michele M, Alfredo G (2012). Cerillo, Stefano Bevilacqua, Danyar Gilmanov, Pierandrea Farneti, Mattia Glauber. Traversing the learning curve in minimally invasive heart valve surgery: a cumulative analysis of an individual surgeon’s experience with a right minithoracotomy approach for aortic valve replacement. Eur J Cardiothorac Surg.

[7] Kouchoukos NT, Wareing TH, Murphy SF, Perrillo JB (1991). Sixteen-year experience with aortic root replacement. Results of 172 operations. Ann Surg.

[8] Prifti E, Bonacchi M, Frati G, Proietti P, Giunti G, Babatasi G (2002). Early and long-term outcome in patients undergoing aortic root replacement with composite graft according to the Bentall’s technique. Eur J Cardiothorac Surg.

[9] Sioris T, David TE, Ivanov J, Armstrong S, Feindel CM (2004). Clinical outcomes after separate and composite replacement of the aortic valve and ascending aorta. J Thorac Cardiovasc Surg.

[10] Etz CD, Bischoff MS, Bodian C, Roder F, Brenner R, Griepp RB (2010). The Bentall procedure: is it the gold standard? A series of 597 consecutive cases. J Thorac Cardiovasc Surg.

[11] Joo HC, Chang BC, Youn YN, Yoo KJ, Lee S (2012). Clinical experience with the Bentall procedure: 28 years. Yonsei Med J.

[12] Kimura N, Tanaka M, Kawahito K, Itoh S, Okamura H, Yamaguchi A (2011). Early- and long-term outcomes after surgery for acute type a aortic dissection in patients aged 45 years and younger. Circ J.

[13] Agg B, Benke K, Szilveszter B, Polos M, Daroczi L, Odler B (2014). Possible extracardiac predictors of aortic dissection in Marfan syndrome. BMC Cardiovasc Disord..

[14] Alpendurada F, Wong J, Kiotsekoglou A, Banya W, Child A, Prasad SK (2010). Evidence for Marfan cardiomyopathy. Eur J Heart Fail.

[15] Judge DP, Dietz HC (2005). Marfan’s syndrome. Lancet.

[16] Murzi M, Cerillo AG, Bevilacqua S, Gilmanov D, Farneti P, Glauber M (2012). Traversing the learning curve in minimally invasive heart valve surgery: a cumulative analysis of an individual surgeon’s experience with a right minithoracotomy approach for aortic valve replacement. Eur J Cardiothorac Surg.

[17] Caputo M, Reeves BC, Rogers CA, Ascione R, Angelini GD (2004). Monitoring the performance of residents during training in off-pump coronary surgery. J Thorac Cardiovasc Surg.

[18] Milano AD, Pratali S, Mecozzi G, Boraschi P, Braccini G, Magagnini E (2003). Fate of coronary ostial anastomoses after the modified Bentall procedure. Ann Thorac Surg.

[19] Kazui T, Yamashita K, Terada H, Washiyama N, Suzuki T, Ohkura K (2003). Late reoperation for proximal aortic and arch complications after previous composite graft replacement in Marfan patients. Ann Thorac Surg.

[20] Gott VL, Greene PS, Alejo DE, Cameron DE, Naftel DC, Miller DC (1999). Replacement of the aortic root in patients with Marfan’s syndrome. N Engl J Med.

[21] Patel ND, Weiss ES, Alejo DE, Nwakanma LU, Williams JA, Dietz HC (2008). Aortic root operations for Marfan syndrome: a comparison of the Bentall and valve-sparing procedures. Ann Thorac Surg.

